# Optimization of Transesterification Reactions with CLEA-Immobilized Feruloyl Esterases from *Thermothelomyces thermophila* and *Talaromyces wortmannii*

**DOI:** 10.3390/molecules23092403

**Published:** 2018-09-19

**Authors:** Anastasia Zerva, Io Antonopoulou, Josefine Enman, Laura Iancu, Peter Jütten, Ulrika Rova, Paul Christakopoulos

**Affiliations:** 1 Division of Chemical Engineering, Department of Civil, Environmental and Natural Resources Engineering, Luleå University of Technology, 97187 Luleå, Sweden; anastasia.zerva@ltu.se (A.Z.); io.antonopoulou@ltu.se (I.A.); Josefine.Enman@ltu.se (J.E.); Ulrika.Rova@ltu.se (U.R.); 2 DuPont Industrial Biosciences, Nieuwe Kanaal 7-S, 6709 PA Wageningen, The Netherlands; Laura.Iancu@dupont.com; 3 Taros Chemicals GmbH & Co. KG, Emil Figge Str 76a, 44227 Dortmund, Germany; pjuetten@taros.de

**Keywords:** feruloyl esterases, transesterification reactions, cross-linked enzyme aggregates, response surface methodology, arabinose ferulate, prenyl ferulate

## Abstract

Feruloyl esterases (FAEs, E.C. 3.1.1.73) are biotechnologically important enzymes with several applications in ferulic acid production from biomass, but also in synthesis of hydroxycinnamic acid derivatives. The use of such biocatalysts in commercial processes can become feasible by their immobilization, providing the advantages of isolation and recycling. In this work, eight feruloyl esterases, immobilized in cross-linked enzyme aggregates (CLEAs) were tested in regard to their transesterification performance, towards the production of prenyl ferulate (PFA) and arabinose ferulate (AFA). After solvent screening, comparison with the activity of respective soluble enzymes, and operational stability tests, FAE125 was selected as the most promising biocatalyst. A central composite design revealed the optimum conditions for each transesterification product, in terms of water content, time, and substrate ratio for both products, and temperature and enzyme load additionally for prenyl ferulate. The optimum product yields obtained were 83.7% for PFA and 58.1% for AFA. FAE125 CLEAs are stable in the optimum conditions of transesterification reactions, maintaining 70% residual activity after five consecutive reactions. Overall, FAE125 CLEAs seem to be able to perform as a robust biocatalyst, offering satisfactory yields and stability, and thus showing significant potential for industrial applications.

## 1. Introduction

Feruloyl esterases (FAEs, E.C. 3.1.1.73) are carboxylic acid hydrolases responsible for the hydrolysis of the ester bond between carboxylic acids (mainly ferulic acid) and sugar moieties of lignocellulosic biomass. During the decomposition of lignocellulose, FAEs act synergistically with xylanases, for the release of hydroxycinnamic acids [1]. The increasing research interest of the past 20 years in the biology of FAEs lies mainly in their ability to release free ferulic acid (FA) from lignocellulosic biomass, and significant efforts have been devoted to the development of a FAE-catalyzed system of FA production from several feedstocks [1]. FA is widely known for its antioxidant properties, but it can also be used as a precursor for the production of several valuable chemicals, mainly vanillin [2], but also guaiacol, catechol, polymers, styrenes and other products [3]. However, FAEs also possess significant biocatalytic potential due to their ability to catalyze (trans)esterification of FA with various substituents, such as alcohols or sugars. Due to the limited solubility of FA in both water- and oil-based media, the production of FA esters can modify the solubility of FA, while maintaining the antioxidant character. Characteristic examples are the production of prenyl ferulate (PFA) by several fungal FAEs in an effort towards the production of more lipophilic, antioxidant compounds from ‘green’ sources, to be applied in the production of cosmeceuticals [4], or the enzymatic synthesis of feruloylated oligosaccharides [5,6]. Besides, feruloylated oligosaccharides (FOs) isolated from natural sources, such as wheat bran and rice, are considered to have health-promoting factors, due to prebiotic activity and activation of dendritic cells, among others [7]. Therefore, they have received approval from the US FDA to be used as additives in food products [8]. Several similar compounds have been synthesized in adequate yields, and tested in regard to their antioxidative activity, prebiotic activity, intracellular ROS modification, and cytotoxicity, with the aim to develop novel cosmeceutical and pharmaceutical formulations [4,5,6]. Moreover, FAEs have been successfully applied to the production of various glucoside esters [9] and alkyl ferulates [10]. Although the product yields obtained from these transesterification attempts are quite satisfactory, in some cases exceeding 70% [6], there are literature data suggesting that product yield can be significantly improved with the use of immobilized biocatalysts [11], simultaneously offering the advantage of biocatalyst recycling.

Cross-linked enzyme aggregates (CLEAS) technology is a well-documented procedure for enzyme immobilization [12,13]. Its many advantages include the simplicity and low cost of the process, as well as the broad applicability of the method. The preparation of CLEAs consists of two steps: the precipitation of the protein molecules from a solution and the cross-linking of the proteins with the use of a suitable bifunctional cross-linker molecule, without the need for a solid support. The resulting CLEAs usually show improved stability and reusability characteristics [14,15], and in some cases, altered specificity has been reported [16]. As a result, the biocatalysts prepared with this methodology usually have all the essential properties for industrial applications.

Eight different FAE preparations have been immobilized in CLEAs in a previous work from our group [17]—namely FAEA1, FAEA2, FAEB1, FAEB2, and *Mt*Fae1a from *Thermothelomyces thermophila*; and FAE68, FAE125, and FAE7262 from *Talaromyces wortmannii*. The objective of the present work was to investigate the synthetic potential of these FAE CLEAs, in two separate transesterification reactions; one for the production of prenyl ferulate and the other for the production of arabinose ferulate (AFA), aiming to optimize the resulting yield.

## 2. Results and Discussion

### 2.1. Effect of Organic Solvents on the Transesterification Performance of FAEs CLEAs

**Figure 1:**
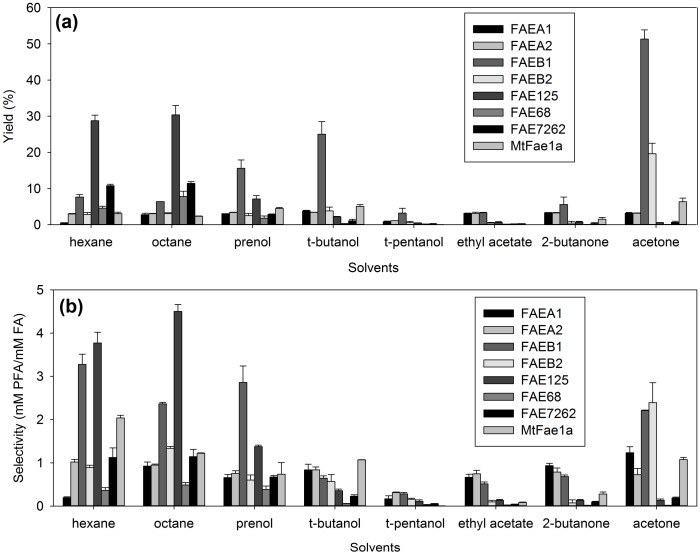
Effect of organic solvents on the synthesis of prenyl ferulate from the obtained CLEAs. (**a**) Product yield (%), (**b**) selectivity.

**Figure 2:**
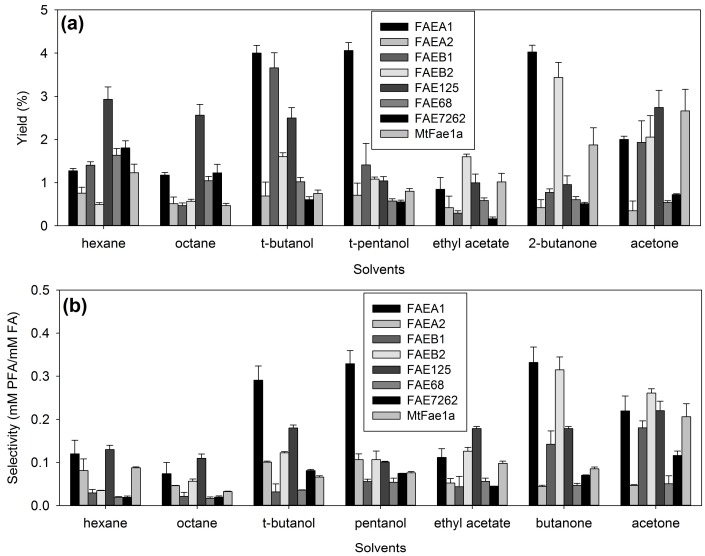
Effect of organic solvents on the synthesis of arabinose ferulate from the obtained CLEAs. (**a**) Product yield (%), (**b**) selectivity.

The first step in the optimization of prenyl and arabinose ferulate synthesis from the obtained CLEAs was the selection of a suitable solvent for the transesterification reactions. For this purpose, a number of solvents with diverse physicochemical characteristics was tested, among them alkanes (hexane and octane), esters (ethyl acetate), alcohols (*t*-butanol, *t*-pentanol), ketones (2-butanone, acetone) and, for the synthesis of prenyl ferulate, prenol was also tested as a solvent. The results of the reactions were evaluated in terms of their target product yield (prenyl ferulate or arabinose ferulate) and selectivity towards hydrolysis of the substrate. The results for prenyl ferulate are shown in Figure 1 and for arabinose ferulate in Figure 2.

Of all the solvents tested, ethyl acetate, *t*-pentanol, 2-butanone, and prenol were found to be the least effective for prenyl ferulate synthesis. Good results were obtained in non-polar solvents, such as hexane and octane, from FAE125 in both reactions, similar to lipase QL from *Alcaligenes* sp. [18]. This preference of FAE125 in non-polar solvents was also true for similar reaction systems with soluble enzyme [19]. Hexane was also the optimum non-polar solvent selected for the synthesis of feruloylated saccharides by *Humicola* spp. Depol 740L [5], together with 2-butanone in microemulsions. The FAEs from *Thermothelomyces thermophila* on the other hand, seem to perform better in polar solvents: The best yield obtained was from FAEB1 in acetone, almost 50% in PFA synthesis. This is a strong indication of the diverse nature of these groups of enzymes. This is also obvious from the results regarding arabinose ferulate synthesis: the best results obtained from FAEs 68, 125, and 7262 were in non-polar solvents, such as alkanes, similar to the respective soluble enzymes [19], while the FAEs A1, A2, B1, B2, and *Mt*Fae1a seem to perform better in alcohols and ketones. However, the yields obtained for arabinose ferulate synthesis were substantially lower: the maximum yield obtained was 4% from FAEA1 in alcohols (*t-*butanol, *t*-pentanol), and 2-butanone, followed by FAEB1 in *t*-butanol (3.7%) and FAEB2 in 2-butanone (3.4%). Surprisingly, in the case of arabinose ferulate synthesis only, FAE125 was found to perform equally well in terms of yield in both non-polar solvents (2.9% and 2.6% in hexane and octane, respectively) and in polar solvents (2.5% and 2.7% in *t*-butanol and acetone respectively). The selected solvents for subsequent experiments were acetone for FAEA1, FAEB1, and FAEB2; octane for FAE125, FAE68, and FAE7262; and *t*-butanol for FAEA2 and *Mt*Fae1a for prenyl ferulate synthesis; and acetone for FAEB1 and *Mt*Fae1a; hexane for FAE125, FAE68, and FAE7262; 2-butanone for FAEA1 and FAEB2; and *t*-butanol for FAEA2 for arabinose ferulate synthesis.

### 2.2. Comparison of the Synthetic Performance of CLEAs with the Corresponding Free Enzymes

**Figure 3:**
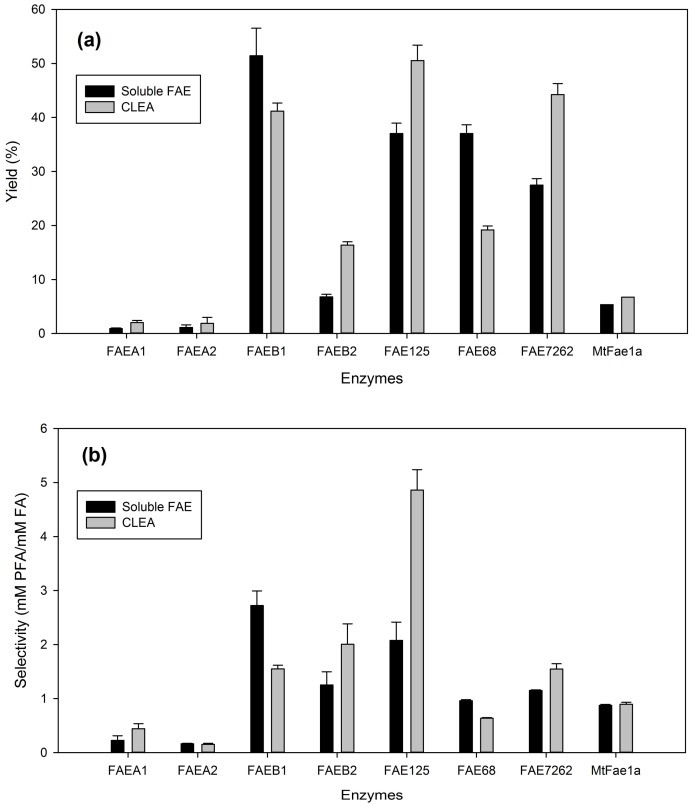
Transesterification performance of the studied CLEAs in prenyl ferulate synthesis, compared to the respective soluble enzymes. (**a**) product yield (%), (**b**) selectivity. The same units of soluble enzymes and CLEAs have been added to each reaction. The reactions were performed in the optimum solvent for each CLEA preparation, as mentioned in Section 2.1.

**Figure 4:**
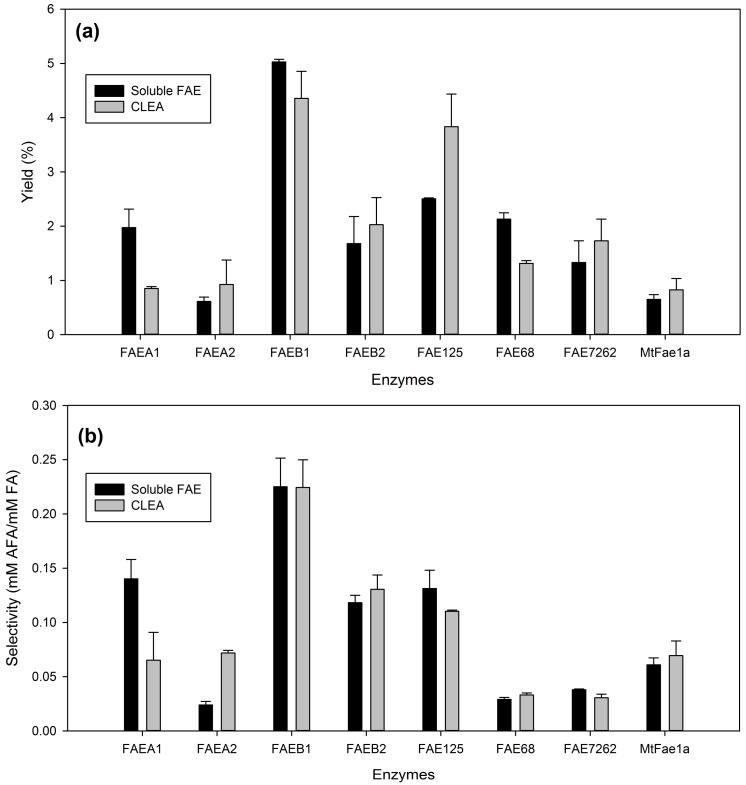
Transesterification performance of the studied CLEAs in arabinose ferulate synthesis, compared to the respective soluble enzymes. (**a**) product yield (%), (**b**) selectivity. The same units of soluble enzymes and CLEAs have been added to each reaction. The reactions were performed in the optimum solvent for each CLEA preparation, as mentioned in Section 2.1.

In order to select a biocatalyst with optimum synthetic activity for the further optimization of the synthetic process, a comparison of the synthetic performance of CLEAs with the corresponding soluble enzymes was carried out, in the best solvent for each CLEA preparation, selected from the previous experiment, adding the same units of soluble and immobilized enzyme for each of the studied FAEs. The results for prenyl ferulate synthesis are shown in Figure 3 and for arabinose ferulate synthesis in Figure 4.

Aside from yield and selectivity, the process metrics for free and immobilized FAEs are presented in Supplementary Materials, Table S1. For prenyl ferulate synthesis, four of the studied FAEs showed considerably higher yield and selectivity in the CLEA form than the corresponding soluble enzymes: FAEA1, FAEB2, FAE125, and FAE7262. The overall performance for synthesis of arabinose ferulate was considerably lower. The only CLEA showing considerably higher yield compared to the respective soluble enzyme was FAE125. The catalyst productivity was also improved for CLEAs of FAEA1, FAEA2, FAE125, FAE7262, and *Mt*Fae1a in PFA synthesis reactions, and for CLEAs of FAEA2, FAEB1, FAE125, FAE7262, and *Mt*Fae1a for AFA synthesis, in respect to the soluble enzymes (Table S1). A slight increase of the reaction rate was also observed for CLEAs of FAEA2, FAEB2, FAE125, FAE7262, and *Mt*Fae1a after immobilization (Table S1). The modification of the enzyme’s properties in the CLEA form can be attributed to their altered conformation and the higher rigidity that usually leads to higher stability. The observed improved transesterification performance of CLEAs with respect to free enzymes has been well documented in the literature, for FAEs [10] as well as lipases [20,21,22]. Overall, FAE125 CLEAs performed best in the synthesis of both PFA (50.5% yield and 4.9 mM PFA/mM FA) and AFA (3.8% yield and 0.11 mM AFA/mM FA), and thus it was selected for further experiments.

### 2.3. Operational Stability of CLEAs in the Synthesis of Prenyl Ferulate in Acetone

**Figure 5:**
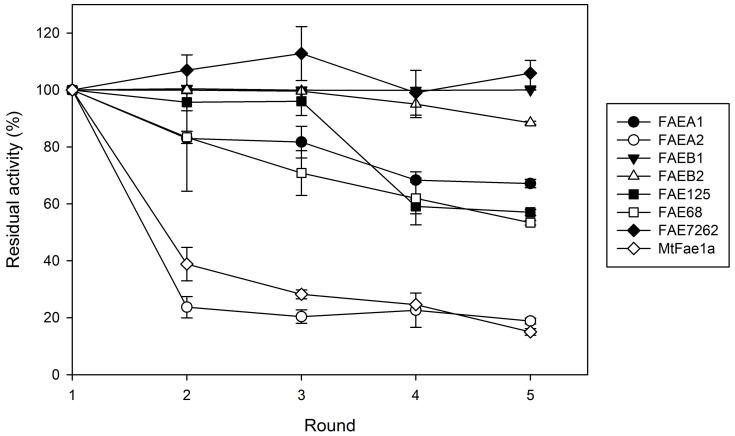
Operational stability of the obtained CLEAs. The residual activity is expressed as a percentage of product yield determined in the first round of reactions.

Reusability is the most important advantage of CLEAs with respect to free enzymes, because it can lower the cost of the catalysts in commercial applications significantly. With a simple centrifugation step, the CLEAs can be removed from the reaction mixture and used again in a new synthetic round. In the present work, we tested the reusability of the FAE CLEAs on the synthesis of prenyl ferulate, in 8-h reactions, and the results are presented in Figure 5.

The most robust enzymes in terms of operational stability were found to be FAEs 7262 and B1, retaining 100% or more of their initial activity. FAE7262 especially shows an enhanced activity in the first rounds of synthesis reactions, which can be attributed to the extensive hours of agitation, leading to shearing of bigger particles into smaller ones, and thus minimizing the diffusion limitations observed in large CLEA particles. FAEs 68, A1, and 125 seem to retain more than 70% of their initial activity in the first three rounds of synthetic reactions, but then their activity drops to slightly above 50%. The least stable FAEs were found to be FAEA2 and *Mt*Fae1a, retaining 18.9% and 15% of their initial activity respectively, after five cycles of synthetic reactions. Previous studies report contradictory results regarding the operational stability properties of immobilized FAEs in transesterification reactions: while the FAE CLEAs previously were found to almost completely lose their synthetic activity after two rounds of butyl ferulate synthesis [10], the immobilization in mesoporous material was shown to lead to more stable enzyme preparations [11]. For butyl ferulate synthesis, the immobilized FAEs were found to maintain over 70% of their initial activity after six rounds of synthetic reactions. These results are in accordance with our data, at least for CLEAs from FAEs 7262, B1, 68, A1, and 125. The satisfactory operational stability obtained from most CLEAs is a great advantage, supporting their use in continuous synthetic processes, for the production and commercialization of high-added value compounds.

### 2.4. Response Surface Methodology for Optimization of Transesterification Yield and Selectivity

Based on all the results above, FAE125 was selected as a candidate CLEA for optimization of several transesterification reaction parameters, for the synthesis of PFA and AFA, due to superior yield and selectivity for both products, as well as satisfying operational stability of the corresponding CLEA. A central composite design was employed for the optimization of prenyl- and arabinose ferulate synthesis. The studied factors were water content, substrate ratio, time, enzyme load and temperature for prenyl ferulate synthesis; and water content, substrate ratio, and time for arabinose ferulate synthesis, all tuned to five levels, as shown in Table 1. In Figure S1 are presented the plots of predicted vs. actual response values for the obtained results for both transesterification reactions.

#### 2.4.1. Prenyl Ferulate Synthesis

The results of the five-factor factorial design for prenyl ferulate synthesis in regard to product yield and selectivity are shown in Table S2. The results of the regression analysis are shown in Table 2. 

The names of the factors are given in Table 1. Concerning the product yield, B, C, D, E, AB, AD, DE, A^2^, C^2^, and E^2^ are statistically significant model terms (*p* < 0.05), while for selectivity, B, C, D, E, AB, CE, DE, A^2^, C^2^, and E^2^ are significant model terms (Table 2).

The responses were fitted to a second-order polynomial equation, neglecting the non-significant model terms (Equation (1) for product yield and Equation (2) for selectivity).

Yield = 61.69 − 0.76A − 8.04B + 4.70C + 7.26D − 11.35E − 5.34AB − 5.40AD + 6.09DE + 2.75A^2^ + 1.55B^2^ − 2.96C^2^ − 2.99E^2^ (1)

Selectivity = 3.68 − 0.025A − 0.51B + 0.27C + 0.39D − 0.71E − 0.23 AB − 0.21CE + 0.24DE + 0.15A^2^ − 0.23C^2^ + 0.12D^2^ − 0.2E^2^ (2)

The equations for both responses appear to be statistically significant at a 99% confidence level (*p* < 0.01). The Model F-values seem to be equally good (11.96 for yield and 10.0 for selectivity), with 0.01% chance that a value this large is due to noise. Lack of fit values were 8.65 for yield and 3.91 for selectivity. *p* > F values for both models are also lower than 0.05, implying that the model terms are statistically significant. The coefficient of determination (R^2^) was calculated 0.89 for yield and 0.88 for selectivity, meaning that the model can explain 89% and 88% of the variability, respectively. Signal to noise ratios also exceed the required minimum 4, and are thus considered adequate for both models (14.4 for yield and 13.8 for selectivity).

**Figure 6:**
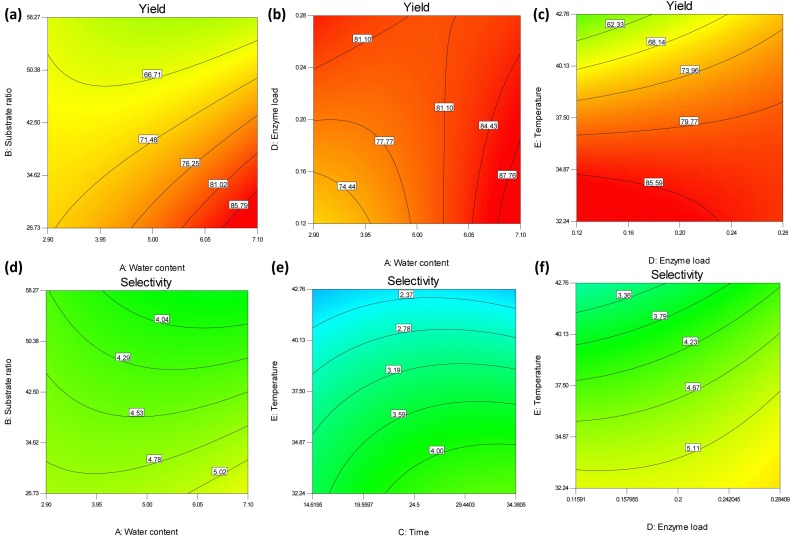
Contour plots for the yield (**a**–**c**) and selectivity (**d**–**f**) of prenyl ferulate synthesis from FAE125 in octane. (**a**,**d**) interaction between substrate ratio and water content, (**b**) interaction between enzyme load and water content, (**d**,**f**) interaction between enzyme load and temperature, (**e**) interaction between temperature and time. In each diagram, all other factors were set to optimum values.

From Equations (1) and (2) and the respective perturbation plots (Figure S2a,b) it is obvious that the most influencing factor for both yield and selectivity of PFA synthesis is temperature, closely followed by substrate ratio. Temperature is a factor that is directly related to the stability of the biocatalyst, while the substrate ratio is of importance in changing the equilibrium of the reaction towards PFA synthesis instead of substrate hydrolysis. For example, one of the most important factors affecting the esterification of ferulic acid with raffinose was also the substrate ratio, in the work performed by Couto et al. [5]. The respective contour plots are presented in Figure 6.

The combination of substrate ratio and water content (Figure 6a) implies that the best yield must be obtained in either high water content or low substrate ratio, at least in the range tested in this study. However, such plots must be interpreted with attention, due to the peculiarities of the FAEs as biocatalysts: low water content is usually expected to favor the transesterification reaction instead of the hydrolysis reaction, but FAEs are not active in total absence of water [23]. The effect of temperature is very intense when it is plotted against enzyme load (Figure 6c,f): at very high temperatures and low enzyme load the yield drops significantly, implying that the effect of temperature is probably due to catalyst instability in high temperatures for long incubation times. According to Design Expert ® Version 11 (Stat-Ease, Minneapolis, MN, USA), the maximum product yield and selectivity can be as high as 83.1% product yield with a selectivity of 5.55 mM PFA/mM FA. The obtained values were similar to those predicted by the algorithm: 83.7% yield and 5.4 mM PFA/mM FA selectivity, which corresponds to 1.47-fold higher yield and 1.51-fold higher selectivity in respect to the central points. The same transesterification reaction with soluble FAE125, in optimized reaction parameters, resulted in only marginally higher yield and selectivity [19]: 87.5% yield and 7.616 selectivity, while in microemulsion systems, the use of FAEB2 resulted in only 71.5% yield, after a step-by-step optimization approach of several reaction parameters, with 2.37 mM PFA/mM FA in regard to selectivity [4].

#### 2.4.2. Arabinose Ferulate Synthesis

In the case of arabinose ferulate synthesis from CLEAs of FAE125, a preliminary five-factor central composite design, similar to the above design for PFA synthesis, was carried out in order to determine the correct upper and lower limits of the selected factors (data not shown). As a result, the enzyme load was set to 0.04 U mL^−1^ reaction and the temperature was set to 32 °C, and a three-factor composite design was implemented, in order to investigate the effect of water content, substrate ratio and time in regard to product yield and selectivity, as shown in Table 1.

The results of the three-factor factorial design for arabinose ferulate synthesis in regard to product yield and selectivity are shown in Table S3. The results of regression analysis are presented in Table 3.

For yield A, C, AC, and A^2^ are significant model terms, while for selectivity, A, AB, AC, BC, and A^2^ are statistically significant model terms (*p* < 0.05) (Table 3). Taking only the statistically significant model terms into consideration, the second-order polynomial equations were (Equation (3) for product yield and Equation (4) for selectivity):

Yield = 41.06 + 16.73 A + 0.052B + 6.44 C +7.27 AC − 10.36 A^2^ (3)

Selectivity = 1.51 + 0.42A − 0.017 B − 9.660E − 003 C + 0.53 AB + 0.54AC + 0.56 BC − 0.28A^2^ (4)

The names of the factors are given in Table 1. The equations for both responses appear to be statistically significant at a 99% confidence level (*p* < 0.01). The model F-values seem to be equally good (7.65 for yield and 22.52 for selectivity), with 0.29% and 0.01% chance that a value this large is due to noise, respectively. Lack of fit values were 13.2 for yield and 114.54 for selectivity. *p* > F values for both models are also lower than 0.05, implying that the model terms are statistically significant. The coefficient of determination (R^2^) was calculated to 0.88 for yield and 0.96 for selectivity, meaning that the model can explain 88% and 96% of the variability, respectively. As the signal to noise ratios are well higher than the minimum 4, they are considered adequate for both models (10.45 for yield and 19.03 for selectivity).

**Figure 7:**
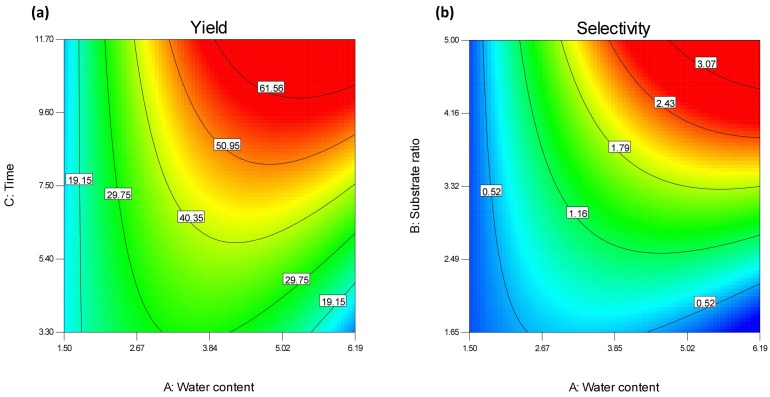
Contour plots for the yield (**a**) and selectivity (**b**) of arabinose ferulate synthesis from FAE125 in hexane. (**a**) Interaction between water content and time, (**b**) interaction between water content and substrate ratio. All other factors were set to their optimum values.

From Equations (3) and (4) and the respective perturbation plots (Figure S2c,d) it is obvious that the most influencing factor for both yield and selectivity of AFA synthesis is the water content of the reaction. In contrast to PFA synthesis, water content seems to be the limiting factor in this case, in order to effectively change the equilibrium of the reaction in favor of AFA synthesis instead of substrate hydrolysis. This conclusion can also be drawn from the contour plots (Figure 7a,b).

From Figure 7 it seems that the best product yield is to be expected at >4% (*v/v*) water content, as lower water content obviously affects FAE activity, while higher water content might shift the equilibrium of the reaction towards hydrolysis. The same is also valid for selectivity (Figure 7b). At the same time, the enhancing effect of water is only obvious in high donor concentrations, otherwise, the reaction equilibrium is shifted towards substrate hydrolysis. This was also true for the transesterification attempts with StFaeC with various sugars [24], where the higher yields were obtained with the lower water content in the tested system, and in the work of Giuliani et al. [25], where pentylferulate synthesis by FAEA from *Aspergillus niger* was decreasing with increasing water content.

The predicted optimal parameters for maximal yield and selectivity, for PFA and AFA synthesis, are presented in Table 4.

The selected model predicted 60.09% yield and 2.83 mM AFA/mM FA selectivity, while the obtained values were 58.14% and 1.48 mM AFA/mM FA respectively, leading to a 1.4-fold increased yield, in comparison to the central points, while selectivity was not significantly improved. Previous studies regarding the optimization of AFA production from VFA, with soluble FAE125, report a maximal yield of 56.2% and selectivity of 1.284, although the adjustment of agitation conditions and dimethyl sulfoxide (DMSO) addition led to even better results [19]. Accordingly, the optimum yield in AFA synthesis by soluble FAEA1 in microemulsion, after optimization of the reaction parameters, was 52.2% and the respective selectivity was 1.12 mM AFA/mM FA [6], while previous studies report a maximum 40% substrate conversion for arabinose ferulate synthesis [24].

### 2.5. Operational Stability of CLEAs in the Optimum Transesterification Conditions

After determining the optimum conditions for synthetic reactions for FAE125 CLEAs, the operational stability was tested again, this time at the optimal conditions. After 5 cycles of consecutive reactions, FAE125 CLEAs were found to maintain more than 70% of their original activity, (73.3 ± 1.3% for PFA synthesis and 70.8 ± 4.3% for AFA synthesis). The productivity (kg product/Unit FAE CLEA) is increased by 445% for PFA synthesis and 339% for AFA synthesis, for five reaction cycles instead of one. These results are very satisfactory, and in accordance with the literature concerning immobilized FAEs [10,11]. For example, in the study by Vafiadi et al. [10] the CLEAs from three different FAE preparations could only be used twice for the synthesis of butyl ferulate, before altogether losing their activity, while Thorn et al. [11] managed to obtain 70% residual activity for the same biotransformation after six rounds of synthetic reactions, but with mesoporous silica-immobilized FAE. Similar reusability properties have also been reported for lipase CLEA [21] in biodiesel production reactions.

### 2.6. Effect of the Transesterification Reactions on the Structure of CLEAs

**Figure 8:**
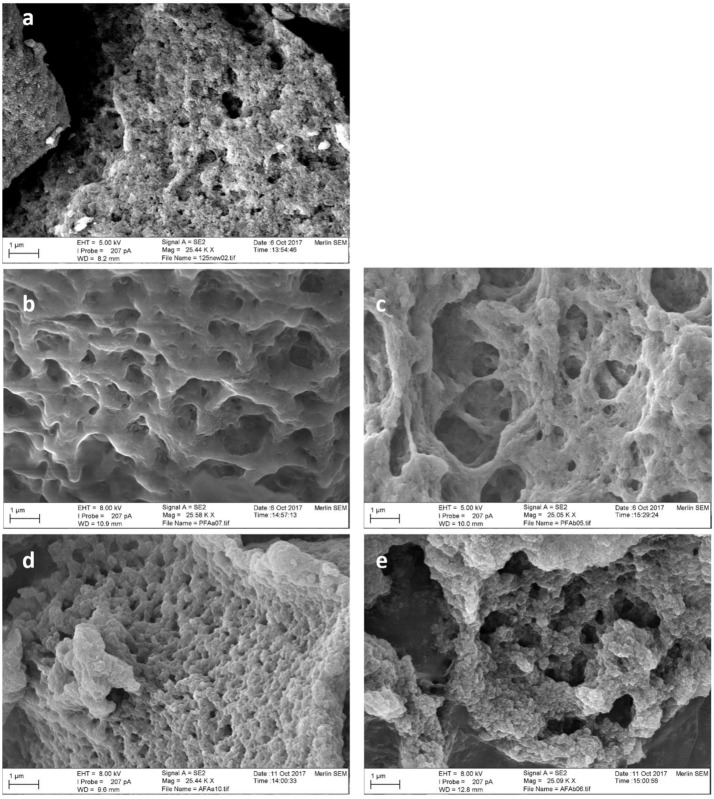
SEM photographs of FAE125 CLEAs before and after use in transesterification reactions, at 25,000×. (**a**) FAE125 CLEAs before use; (**b**) FAE125 CLEAs after one round of PFA synthesis; (**c**) FAE125 CLEAs after two rounds of PFA synthesis; (**d**) FAE125 CLEAs after one round of AFA synthesis; (**e**) FAE125 CLEAs after two rounds of AFA synthesis.

FAE125 CLEAs were analyzed with SEM before and after use in transesterification reactions with both substrates, in optimum conditions derived from the previous experiments, and the resulting photographs are shown in Figure 8.

From Figure 8 it is obvious that the prolonged reaction time of PFA synthesis in optimum conditions (34.38 h) substantially changes the microscopic structure of the CLEAs, making them form clusters of bigger size, resulting in a more uniform and round shape, compared to the initial small aggregates of approximately 0.1 μm. To a lesser extent, this is also true for the microscopic structure of CLEAs after AFA synthesis reactions, but the appearance of the aggregates is more similar to the original CLEA, although they are slightly larger in size. These results imply that the AFA synthesis reactions are much less damaging to the CLEA structure, compared to PFA synthesis, which can be expected due to largely different incubation times (34.38 h for PFA synthesis and 10.9 h for AFA).

## 3. Materials and Methods

### 3.1. Materials

VFA (vinyl ferulate) was provided by Taros Chemicals GmbH & Co. KG (Dortmund, Germany). Methyl ferulate (MFA) was purchased from Alfa Aesar (Karlsruhe, Germany), while FA, prenol (99%), *n*-hexane (<0.02% water), *t*-butanol (anhydrous, ≥99.5%), MOPS solution 1 M, and other materials were purchased from Sigma-Aldrich (Saint Louis, MO, USA).

### 3.2. Enzymes

The genes encoding FAEA1, FAEA2, FAEB1, and FAEB2 of *T. thermophila* C1 were homologously over-expressed in proper C1 strains with low cellulase production [26,27]. The DNA sequences encoding the putative FAE68, FAE7262, and FAE125 from the genome of *T. wortmannii*, and the respective genes were synthesized, (GeneArt, Schwerte, Germany) and cloned in a transformation vector as previously described [27]. The respective vectors, including a suitable promoter and a selection marker were transformed to a suitable C1 strain. The production of heterologous FAEs was performed in fed-batch aerobic cultures, in minimal medium supplemented with trace elements [28]. The culture supernatant was concentrated and dialyzed against 10 mM phosphate buffer pH 6.5. The resulting enzyme preparation was freeze-dried for long-term storage. The FAE *Mt*Fae1a, corresponding to the amino acid sequence of FAEB2 was expressed in *Pichia pastoris* as described previously [29]. The GenBank IDs for all the FAEs used in this work, is as follows: FAEA1, JF826027.1; FAEA2, JF826028.1; FAEB1, Patent Sequence ID 71; FAEB2, JF826029.1; *Mt*Fae1a, AEO62008.1; FAE68, MF362596.1; FAE125, MF362595.1; FAE7262, MF362597.1 [17].

### 3.3. Enzyme Activities

FAEs hydrolytic activity was measured using methyl ferulate as the substrate. Enzyme samples were incubated in MOPS-NaOH buffer pH 6, 100 mM with 1 mM MFA for 10 min in 45 °C and 1300 rpm in an Eppendorf Thermomixer comfort (Eppendorf, Hamburg, Germany). The reaction was terminated with a 10 min incubation in 100 °C. The amount of released ferulic acid was quantified with HPLC, as previously described [30], using the appropriate standard curves. 1 Unit of enzyme activity corresponds to the amount of enzyme releasing 1 μmol of free ferulic acid per minute in the above conditions.

### 3.4. Transesterification Reactions

Freeze-dried CLEA samples or free enzymes were added to a mixture of 96.8:3.2 *v*/*v* solvent: 100 mM MOPS-NaOH pH 6, with 50 mM vinyl ferulate as a donor, to a total volume of 250 μL, unless otherwise stated. For prenyl ferulate synthesis, 200 mM prenol was introduced in the reaction, while for arabinose ferulate synthesis, 30 mM arabinose was used. For arabinose ferulate synthesis reactions, 5% (*v*/*v*) DMSO was also added, in order to facilitate arabinose solubilization in the reaction mixture. The transesterification reactions were routinely performed at 45 °C and 1300 rpm for 8 h in an Eppendorf Thermomixer, unless otherwise stated. Reactions were terminated with a 50-fold dilution in acetonitrile, and subsequently analyzed with HPLC. For PFA synthesis reactions, HPLC analysis was performed as previously described [31], using the appropriate standard curves. For AFA synthesis, a linear gradient from 0 to 100% acetonitrile was used for the proper separation of AFA and FA [31].

### 3.5. CLEA Preparations

The FAE preparations were immobilized to CLEAs as previously described [10], following the procedure of Schoevaart et al. [32]. Briefly, 10 μL of enzyme solution was added to 90 μL of a proper precipitant, followed by 15 min incubation in room temperature and 1300 rpm. Then, 5 μL of glutaraldehyde solution was added to the mixture, and the immobilization was left to proceed for 3 h in room temperature and 1300 rpm. The mixture was quenched in 900 μL 100 mM MOPS-NaOH buffer pH 6, and centrifuged. The precipitated CLEAs were washed twice with MOPS-NaOH buffer 100 mM, pH 6, to remove unreacted enzyme and glutaraldehyde. Finally, the CLEAs were dispersed in 1 mL MOPS-NaOH buffer, and an aliquot was withdrawn and freeze-dried for subsequent use in transesterification reactions. The residual activity of CLEAs in respect to the enzyme solutions used is presented in Table S1.

### 3.6. Central Composite Design

A factorial, central composite design was constructed in order to determine the effect of five factors on the transesterification performance of FAE 125 for PFA and three factors on the transesterification performance of FAE 125 for AFA. The studied factors were water content, substrate ratio, time, enzyme load, and temperature for prenyl ferulate synthesis, and water content, substrate ratio and time for arabinose ferulate synthesis, all tuned to five levels, as shown in Table 1. The total reaction volume was 200 μL, and the water phase in the reactions was inserted as MOPS-NaOH 100 mM buffer, pH 6. The vinyl ferulate concentration was kept constant at 60 mM for prenyl ferulate synthesis and 80 mM for arabinose ferulate synthesis. The central composite design for prenyl ferulate synthesis included 50 reactions, 8 of which were replicates of the central points, 10 reactions for axial points, and 32 reactions for the factorial design (Table S2), while for arabinose ferulate synthesis, the design included 20 reactions, 6 of which were replicates of the central points, 6 reactions for axial points, and 8 reactions for the factorial design (Table S3). The responses selected in each case were the yield and the selectivity towards the desired product.

### 3.7. Statistical Analysis

The regression analysis of the experimental data and the response surface plots were performed with the software platform Design-Expert, Stat-Ease, Inc., Minneapolis, MN, USA. The statistical parameters were estimated with ANOVA.

### 3.8. Structure of CLEAs

For the scanning electron microscopy (SEM) analysis the CLEA samples were first coated with a thin layer of gold. The structural analysis of these samples was then performed with a high-resolution field-emission gun scanning electron microscope, Zeiss Merlin, fitted with an Oxford Instruments large-area energy dispersive spectrometer (FEG-SEM-EDS), using an accelerating voltage between 5.0–8.0 kV and a beam current of 207 pA.

### 3.9. Operational Stability of CLEAs

The reusability of all CLEAs in the synthesis of PFA was tested with five consecutive cycles of transesterification reactions in acetone, 45 °C, for 8 h, of 1 mL total volume. After each cycle, CLEAs were centrifuged off and washed with acetone and MOPS-NaOH buffer, to remove unreacted substrates.

The reusability of FAE125 CLEAs after the optimization of the transesterification reaction parameters was performed with five consecutive cycles of transesterification reaction in the optimum conditions selected for each synthetic reaction. After each cycle, CLEAs were centrifuged off and washed with acetone and MOPS-NaOH buffer to remove unreacted substrates.

The residual transesterification activity was expressed as a percentage of conversion yield obtained in each cycle.

## 4. Conclusions

Overall, the results of the present work support that, in some cases, the immobilization of fungal FAEs in CLEA form can improve the transesterification performance of these enzymes. After careful optimization of several reaction parameters, the yield and selectivity towards the desired product can be significantly enhanced, while maintaining superior reusability characteristics. These properties support the feasibility of the industrial application of these reactions, in large scale.

## Figures and Tables

**Table 1:** Variables and their levels for the central composite experimental design for PFA and AFA synthesis.

Prenyl ferulate synthesis	**Factor**	**Name**	**Units**	**Minimum**	**Maximum**	**−1**	**+1**	**Mean**	**Std. Dev.**
	A	Water content	% *v*/*v*	0.00	10.00	2.90	7.10	5.00	1.98
	B	Substrate ratio	mM prenol/mM VFA	5.00	80.00	26.73	58.27	42.50	14.82
	C	Time	h	1.00	48.00	14.62	34.38	24.50	9.29
	D	Enzyme load	U/mL	0.00	0.40	0.12	0.28	0.20	0.08
	E	Temperature	°C	25.00	50.00	32.24	42.76	37.50	4.94
Arabinose ferulate synthesis	**Factor**	**Name**	**Units**	**Minimum**	**Maximum**	**−1**	**+1**	**Mean**	**Std. Dev.**
	A	Water content	% *v*/*v*	0.31	6.19	1.50	5.00	3.25	1.52
	B	Substrate ratio	mM arabinose/mM VFA	1.65	5.85	2.50	5.00	3.75	1.09
	C	Time	h	3.30	11.70	5.00	10.00	7.50	2.18

**Table 2:** Results of the regression analysis of the central composite design for PFA synthesis.

Source	Response 1: Yield			Response 2: Selectivity		
	Mean Square	F Value	Probability > F	Mean Square	F Value	Probability > F
Model	830.41	11.95	<0.0001	2.87	10.00	<0.0001
A-Water content	24.88	0.36	0.5543	0.03	0.10	0.7574
B-Substrate ratio	2801.62	40.33	<0.0001	11.19	39.02	<0.0001
C-Time	956.78	13.77	0.0009	3.16	11.02	0.0025
D-Enzyme load	1443.55	20.78	<0.0001	5.19	18.09	0.0002
E-Temperature	5578.21	80.30	<0.0001	21.72	75.74	<0.0001
AB	913.54	13.15	0.0011	1.63	5.67	0.0243
AC	6.49	0.09	0.7622	0.03	0.10	0.7491
AD	934.61	13.45	0.0010	0.38	1.31	0.2619
AE	1.34	0.02	0.8904	0.00	0.00	0.9663
BC	8.68	0.12	0.7264	0.19	0.65	0.4257
BD	1.22	0.02	0.8953	0.01	0.04	0.8509
BE	80.35	1.16	0.2914	0.49	1.73	0.1996
CD	12.55	0.18	0.6740	0.08	0.29	0.5953
CE	129.43	1.86	0.1831	1.43	4.99	0.0336
DE	1187.75	17.10	0.0003	1.90	6.62	0.0157
A^2^	495.59	7.13	0.0125	1.35	4.70	0.0388
B^2^	131.07	1.89	0.1805	0.12	0.42	0.5237
C^2^	399.16	5.75	0.0234	2.79	9.72	0.0042
D^2^	218.38	3.14	0.0871	0.48	1.68	0.2053
E^2^	409.62	5.90	0.0218	2.14	7.45	0.0108
Lack of Fit	89.19	8.65	0.0035	0.35	3.91	0.0355

**Table 3:** Results of the regression analysis of the central composite design for ΑFA synthesis.

Source	Response 1: Yield			Response 2: Selectivity		
	Mean Square	F Value	Probability > F	Mean Square	F Value	Probability > F
Model	653.14	7.65	0.0029	0.75	22.52	<0.0001
A-Water content	3466.99	40.58	0.0001	1.98	59.53	<0.0001
B-Substrate ratio	9.75	0.11	0.7432	0.00	0.06	0.8107
C-Time	599.00	7.01	0.0266	0.00	0.01	0.9196
AB	79.66	0.93	0.3595	1.67	50.19	<0.0001
AC	446.82	5.23	0.0480	1.72	51.60	<0.0001
BC	119.53	1.40	0.2672	1.88	56.57	<0.0001
A^2^	1503.48	17.60	0.0023	1.08	32.28	0.0003
B^2^	5.36	0.06	0.8078	0.01	0.16	0.7018
C^2^	79.55	0.93	0.3598	0.02	0.54	0.4830
Lack of Fit	175.59	13.20	0.01	0.07	111.54	<0.0001

**Table 4:** Overview of optimum conditions and the obtained results of the present study for both products tested.

Summary of the Obtained Results	PFA Synthesis	AFA Synthesis
**Optimized conditions**		
Water content (%)	7.1	5.0
Substrate ratio (mM acceptor/mM VFA)	26.7	5.0
Enzyme concentration (U/mL)	0.116	0.04
Temperature (°C)	32	32
Time (h)	34.38	10.0
**Predicted responses**		
Product yield (%)	83.1	60.09
Selectivity (mM product/mM FA)	5.6	2.83
**Obtained parameters**		
Product concentration (mM)	50.25 ± 0.48	46.15 ± 1.4
Product yield (%)	83.74 ± 0.8	58.14 ± 1.75
Overall yield (%)	99.32 ± 0.1	97.47 ± 0.9
Rate (mol product/ U FAE L h)	74.46 ± 0.7	140.95 ± 4.24
Selectivity (mM product /mM FA)	5.38 ± 0.29	1.48 ± 0.08

## Supplementary Materials

The following are available online. Table S1. Metrics of AFA and PFA synthetic reactions, with free and immobilized FAEs. Supplementary File 1: Final equations in terms of actual factors for the yield and selectivity of PFA and AFA, including all model terms. Table S2: Experimental design and results of central composite design for PFA synthesis. Table S3: Experimental design and results of central composite design for AFA synthesis. Figure S1: Plots of the predicted vs actual response values for (a,c) yield (%) and (b,d) selectivity (mM PFA or AFA/mM FA) for prenyl ferulate synthesis (a,b) and arabinose ferulate synthesis (c,d). Figure S2: Perturbation plots for prenyl ferulate synthesis (a,b) and arabinose ferulate synthesis (c,d), presenting the effect of each variable on the (a,c) yield and (b,d) selectivity of the reaction.
