# Supplementary material for: Optimization of Transesterification Reactions with CLEA-Immobilized Feruloyl Esterases from Thermothelomyces thermophila and Talaromyces wortmannii
Source: Molecules. 2018 Sep 19;23(9):2403. doi: 10.3390/molecules23092403 (PMC6225245; doi:10.3390/molecules23092403)
Supplement: Supplementary file 1 [file molecules-23-02403-s001.pdf]

molecules

Supplementary material

Optimization of Transesterification Reactions with CLEA-Immobilized Feruloyl Esterases from *Thermothelomyces thermophila* and *Talaromyces wortmannii*

Anastasia Zerva [1], Io Antonopoulou [1], Josefine Enman [1], Laura Iancu [2], Peter Jütten [3], Ulrika Rova [1], and Paul Christakopoulos [1,*]

[1] Division of Chemical Engineering, Department of Civil, Environmental and Natural Resources Engineering, Luleå University of Technology, 97187 Luleå, Sweden; anastasia.zerva@ltu.se (A.Z.); io.antonopoulou@ltu.se (I.A.); Josefine.Enman@ltu.se (J.E.); Ulrika.Rova@ltu.se (U.R.)
[2] DuPont Industrial Biosciences, Nieuwe Kanaal 7-S, 6709 PA Wageningen, The Netherlands; Laura.Iancu@dupont.com
[3] Taros Chemicals GmbH & Co. KG, Emil Figge Str 76a, 44227 Dortmund, Germany; pjuetten@taros.de
* Correspondence: paul.christakopoulos@ltu.se; Tel.: +46-920-492510

Table S1. Metrics of AFA and PFA synthetic reactions, with free and immobilized FAEs. Residual activity: FAE activity of CLEAs after immobilization; Effectiveness ratio: rate with immobilized enzyme/ rate with free enzyme; Productivity: g product/ Unit; Enzyme consumption: Unit/g product.

		Effectiveness ratio		Enzyme consumption				Productivity			
				Free enzymes		CLEAs		Free enzymes		CLEAs	
	Residual activity (%) [17]	PFA	AFA	PFA	AFA	PFA	AFA	PFA	AFA	PFA	AFA
FAEA1	28.95	2.2	0.4	1642.8	620.4	756.3	1437.8	0.00061	0.0016	0.00132	0.0007
FAEA2	5.9	1.7	1.5	1360.0	1999.2	811.1	1323.1	0.00074	0.0005	0.00123	0.0008
FAEB1	49.64	0.8	0.9	29.6	243.9	37.0	281.51	0.034	0.004	0.03	0.0035
FAEB2	68.87	2.4	1.2	224.3	730.4	93.1	604.5	0.0045	0.0013	0.01	0.0016
FAE125	15.15	1.1	1.5	28.7	489.2	27.0	319.7	0.035	0.002	0.037	0.003
FAE68	98.31	0.4	0.6	20.3	575.8	49.0	932.4	0.049	0.0017	0.02	0.001
FAE7262	30.85	1.7	1.3	38.2	920.0	22.4	708.5	0.026	0.001	0.045	0.0014
MtFae1a	49.13	1.3	1.3	285.1	1883.1	225.7	1482.9	0.0035	0.0005	0.0044	0.0007

Supplementary file 1. Final equations in terms of actual factors for the yield and selectivity of PFA and AFA, including all model terms.

PFA

Yield = -7.58 +5.96 *Water content +0.43* Substrate ratio +3.18* Time -410.32*Enzyme load +4.36* Temperature -0.16* Water content * Substrate ratio +0.02 *Water content*Time -30.57 Water content * Enzyme load -0.02* Water content* Temperature +3.34E-003 * Substrate ratio * Time -0.15 * Substrate ratio * Enzyme load -0.02 * Substrate ratio * Temperature -0.75 * Time * Enzyme load -0.040 * Time * Temperature +13.79* Enzyme load * Temperature +0.68* Water content^2 +6.22E-003 * Substrate ratio^2 -0.03* Time^2 +372.85 * Enzyme load^2 -0.1* Temperature^2

Selectivity = -0.97 -4.98E-003* Water content -0.056 * Substrate ratio +0.32* Time -17.85* Enzyme load +0.33* Temperature -6.8E-003 * Water content * Substrate ratio +1.47E-003 * Water content * Time -0.61 * Water content * Enzyme load +3.65E-004 * Water content * Temperature -4.91E-004 * Substrate ratio * Time -0.01 * Substrate ratio * Enzyme load +1.5E-003 * Substrate ratio * Temperature -0.06 * Time * Enzyme load -4.07E-003 * Time * Temperature +0.55* Enzyme load * Temperature +0.035* Water content^2 +1.88E-004 *Substrate ratio^2-2.3E-003* Time^2 +17.52* Enzyme load^2 -7.15E-003* Temperature^2

AFA

Yield = +33.86 +10.67* Water content -12.7* Substrate ratio -3.32 Time +1.7 Water content * Substrate ratio +2.01* Water content * Time +1.46 Substrate ratio* Time -3.4* Water content^2 -0.4 *Substrate ratio^2 – 0.4 *Time^2

Selectivity = +11.46 – 1.02* Water content -2.28* Substrate ratio -1.18* Time +0.24* Water content * Substrate ratio +0.12* Water content * Time +0.18* Substrate ratio * Time -0.09* Water content^2 +0.01* Substrate ratio^2 +5.72452E-003 * Time^2

Table S2. Experimental design and results of central composite design for PFA synthesis. [a]: central values.

Standard order	Water content (% v/v)	Substrate ratio (mM prenol/mM VFA)	Time (h)	Enzyme load (U/mL)	Temperature (°C)	Response			
						Yield (%)		Selectivity (mM PFA/mM FA)	
						Actual	Predicted	Actual	Predicted
1	2.90	26.73	14.62	0.12	32.24	69.71	55.45	4.29	3.55
2	7.10	26.73	14.62	0.12	32.24	80.06	74.94	4.54	4.09
3	2.90	58.27	14.62	0.12	32.24	58.06	52.57	3.11	2.92
4	7.10	58.27	14.62	0.12	32.24	53.72	50.68	3.12	2.57
5	2.90	26.73	34.38	0.12	32.24	83.28	68.18	5.99	4.70
6	7.10	26.73	34.38	0.12	32.24	81.92	89.47	4.74	5.37
7	2.90	58.27	34.38	0.12	32.24	76.89	67.38	3.91	3.77

8	7.10	58.27	34.38	0.12	32.24	71.52	67.30	3.89	3.54
9	2.90	26.73	14.62	0.28	32.24	78.67	75.09	4.63	4.57
10	7.10	26.73	14.62	0.28	32.24	74.66	72.96	4.67	4.68
11	2.90	58.27	14.62	0.28	32.24	76.36	71.42	4.02	3.87
12	7.10	58.27	14.62	0.28	32.24	52.06	47.92	3.25	3.09
13	2.90	26.73	34.38	0.28	32.24	81.16	85.31	4.97	5.52
14	7.10	26.73	34.38	0.28	32.24	86.64	84.99	6.91	5.76
15	2.90	58.27	34.38	0.28	32.24	78.74	83.74	4.49	4.52
16	7.10	58.27	34.38	0.28	32.24	68.94	62.04	3.82	3.86
17	2.90	26.73	14.62	0.12	42.76	29.42	28.17	1.95	1.81
18	7.10	26.73	14.62	0.12	42.76	55.45	46.84	2.82	2.37
19	2.90	58.27	14.62	0.12	42.76	26.81	18.95	1.62	1.68
20	7.10	58.27	14.62	0.12	42.76	27.46	16.25	1.85	1.34
21	2.90	26.73	34.38	0.12	42.76	32.41	32.86	2.15	2.12
22	7.10	26.73	34.38	0.12	42.76	61.27	53.33	3.51	2.80
23	2.90	58.27	34.38	0.12	42.76	31.01	25.72	1.88	1.68
24	7.10	58.27	34.38	0.12	42.76	26.02	24.82	1.75	1.47
25	2.90	26.73	14.62	0.28	42.76	77.95	72.18	4.31	3.81
26	7.10	26.73	14.62	0.28	42.76	66.30	69.23	3.99	3.94
27	2.90	58.27	14.62	0.28	42.76	70.40	62.18	3.76	3.61
28	7.10	58.27	14.62	0.28	42.76	33.85	37.85	2.55	2.84
29	2.90	26.73	34.38	0.28	42.76	77.83	74.36	3.78	3.92
30	7.10	26.73	34.38	0.28	42.76	72.98	73.21	4.08	4.17
31	2.90	58.27	34.38	0.28	42.76	72.48	66.44	3.73	3.41
32	7.10	58.27	34.38	0.28	42.76	35.08	43.92	2.29	2.76
33	0.00	42.50	24.50	0.20	37.50	60.12	80.44	4.24	4.80
34	10.00	42.50	24.50	0.20	37.50	77.11	76.83	4.13	4.68
35	5.00	5.00	24.50	0.20	37.50	80.97	89.50	4.35	5.33

36	5.00	80.00	24.50	0.20	37.50	39.74	51.25	2.78	2.91
37	5.00	42.50	1.00	0.20	37.50	16.09	35.19	1.12	1.94
38	5.00	42.50	48.00	0.20	37.50	56.59	57.54	2.93	3.22
39	5.00	42.50	24.50	0.00	37.50	0.26	25.18	0.02	1.50
40	5.00	42.50	24.50	0.40	37.50	76.12	71.25	4.62	4.25
41	5.00	42.50	24.50	0.20	25.00	60.50	73.16	3.52	4.42
42	5.00	42.50	24.50	0.20	50.00	11.79	19.17	0.85	1.06
43 [a]	5.00	42.50	24.50	0.20	37.50	59.75	58.30	3.73	3.66
44 [a]	5.00	42.50	24.50	0.20	37.50	57.21	58.30	3.52	3.66
45 [a]	5.00	42.50	24.50	0.20	37.50	54.53	58.30	4.04	3.66
46 [a]	5.00	42.50	24.50	0.20	37.50	57.41	58.30	3.68	3.66
47 [a]	5.00	42.50	24.50	0.20	37.50	53.09	58.30	3.13	3.66
48 [a]	5.00	42.50	24.50	0.20	37.50	61.12	58.30	3.26	3.66
49 [a]	5.00	42.50	24.50	0.20	37.50	58.17	58.30	3.78	3.66
50 [a]	5.00	42.50	24.50	0.20	37.50	51.93	58.30	3.40	3.66

Table S3. Experimental design and results of central composite design for AFA synthesis. [a]: central values.

Standard order	Water content (% v/v)	Substrate ratio (mM arabinose/ mM VFA)	Time (h)	Response			
				Yield (%)		Selectivity (mM PFA/mM FA)	
				Actual	Predicted	Actual	Predicted
1	1.5	2.5	5	14.62	14.48	2.62	2.02
2	5	2.5	5	35.97	35.51	1.35	1.43
3	1.5	5	5	1.43	10.63	0.21	0.51
4	5	5	5	33.46	31.25	1.29	1.04
5	1.5	2.5	10	1.13	13.26	0.17	0.51
6	5	2.5	10	53.44	54.16	1.29	1.07
7	1.5	5	10	1.95	12.34	0.25	0.25
8	5	5	10	42.78	52.83	1.22	1.90
9	0.31	3.75	7.5	0.37	-13.63	0.07	0.09
10	6.19	3.75	7.5	38.15	38.11	1.12	0.99
11	3.25	1.65	7.5	44.50	42.00	1.37	1.66
12	3.25	5.85	7.5	49.16	37.64	1.50	1.10
13	3.25	3.75	3.3	25.27	26.26	1.36	1.69
14	3.25	3.75	11.7	58.40	43.38	1.60	1.16
15 [a]	3.25	3.75	7.5	43.84	43.84	1.51	1.48
16 [a]	3.25	3.75	7.5	45.42	43.84	1.44	1.48
17 [a]	3.25	3.75	7.5	44.41	43.84	1.46	1.48
18 [a]	3.25	3.75	7.5	36.11	43.84	1.49	1.48
19 [a]	3.25	3.75	7.5	45.42	43.84	1.48	1.48
20 [a]	3.25	3.75	7.5	45.42	43.84	1.48	1.48

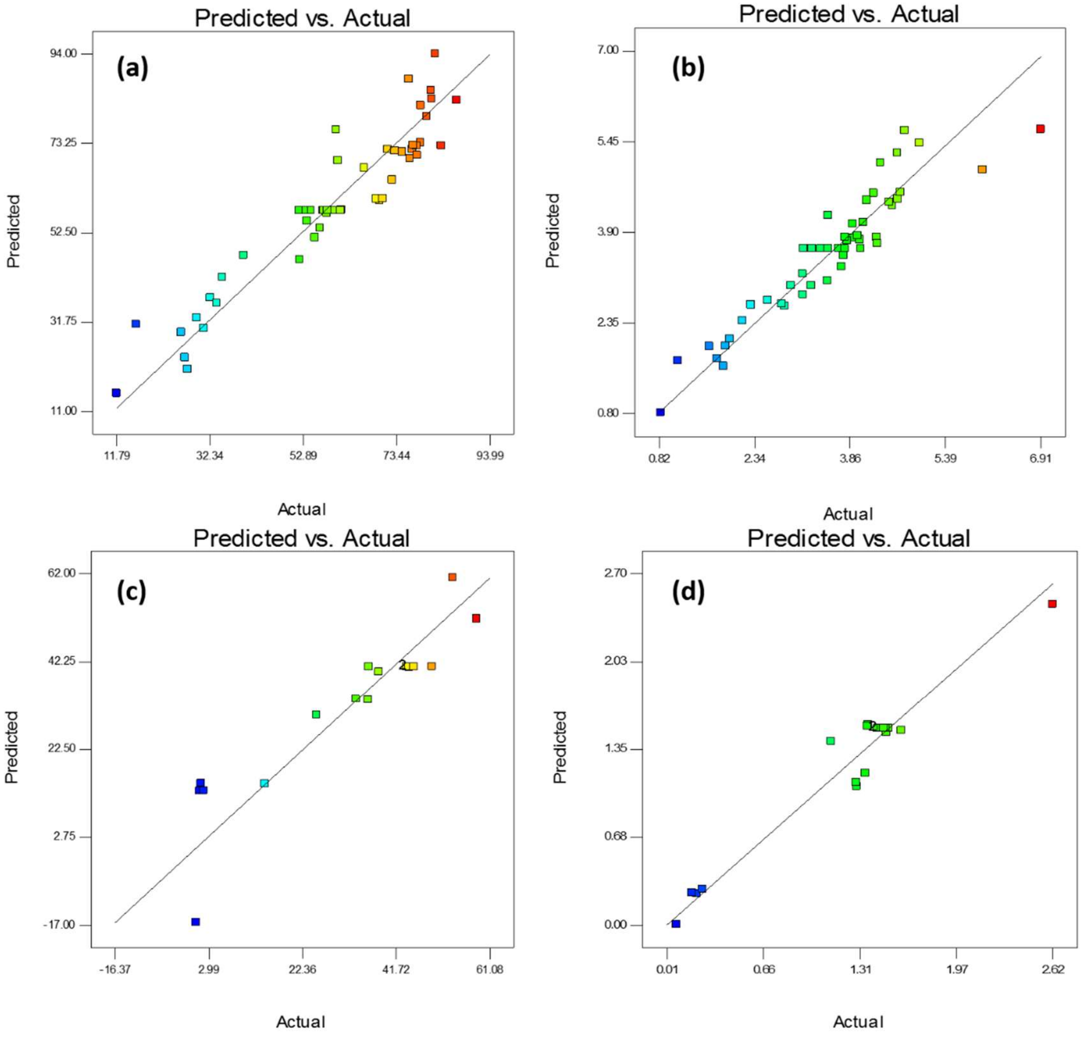

Figure S1. Plots of the predicted vs actual response values for (a, c) yield (%) and (b, d) selectivity (mM PFA or AFA/mM FA) for prenyl ferulate synthesis (a, b) and arabinose ferulate synthesis (c, d).

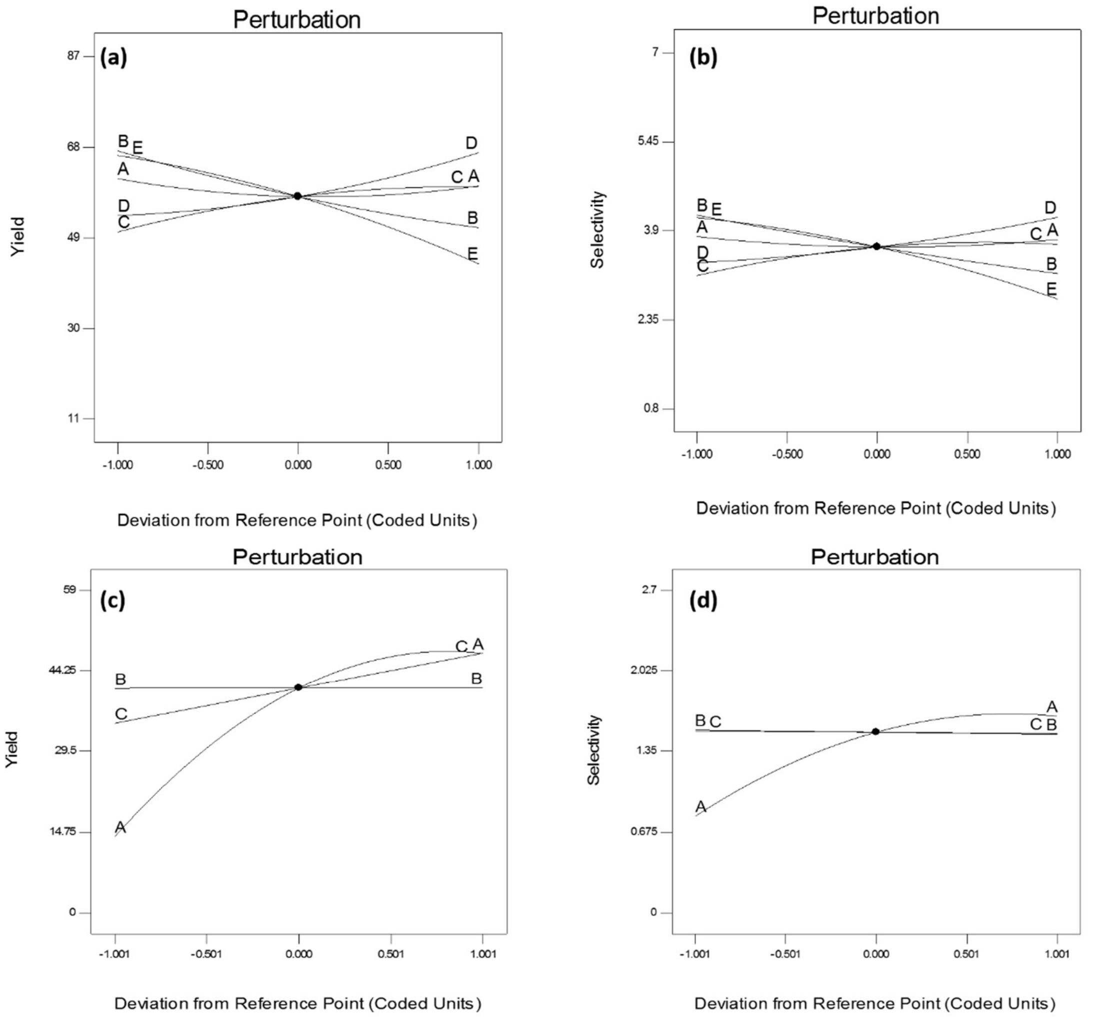

Figure S2. Perturbation plots for prenyl ferulate synthesis (a, b) and arabinose ferulate synthesis (c, d), presenting the effect of each variable on the (a, c) yield and (b, d) selectivity of the reaction. A: water content, B: substrate ratio, C: time, D: Enzyme load, E: temperature.
